# Post-anesthesia care unit desaturation in adult deep extubation patients

**DOI:** 10.1186/s13104-021-05560-5

**Published:** 2021-04-20

**Authors:** Jeremy Juang, Martha Cordoba, Mark Xiao, Alex Ciaramella, Jeremy Goldfarb, Jorge Enrique Bayter, Alvaro Andres Macias

**Affiliations:** 1 Department of Anesthesiology, Massachusetts Eye and Ear, 243 Charles St, Boston, MA 02114 USA; 2 Harvard Medical School, Boston, MA 20114 USA; 3 Director Medico, Km 2 Anillo Vial Floridablanca - Girón, Clinica El PinarEcoparque Empresarial Natura Torre 2 piso 1 y 2, Piedecuesta, Colombia

**Keywords:** Deep extubation, Respiratory pathology, Anesthesia, Ambulatory surgery, PACU workflow, Desaturation

## Abstract

**Objective:**

Deep extubation refers to endotracheal extubation performed while a patient is deeply anesthetized and without airway reflexes. After deep extubation, patients are sent to the post-anesthesia care unit (PACU) to recover, an area with notably different management and staffing than the operating room (OR). One of the most frequent and concerning complications to occur in the PACU is hypoxemia. As such, this study seeks to evaluate the incidence of desaturation, defined by SpO2 < 90% for longer than 10 s, in the PACU following deep extubation. Additionally, we hope to assess the consequence of desaturation on perioperative workflow by comparing PACU recovery times.

**Results:**

Following deep extubation, 4.3% of patients (13/300) experienced desaturation in the PACU. Every episode was notably minor, with patients reverting to normal saturation levels within a minute. Of the 26 case factors assessed, 24 had no significant association desaturation in the PACU, including the amount of time spent in the PACU. History of asthma was the only statistically significant factor found to be positively associated with desaturation. We find that PACU desaturation episodes following deep extubation are rare. Our findings suggest that deep extubation is a viable and safe option for patients without significant respiratory tract pathology.

## Introduction

Compared to traditional endotracheal extubation, deep extubation can be advantageous because of its potential for a "smooth" emergence from anesthesia. Patients, without return of their airway reflexes, are less inclined to buck or cough. This is particularly important in procedures requiring the maintenance of stable intraocular and intracranial pressures, such as in neurologic, ophthalmic, and head-and-neck surgery [1]. After extubation, patients are sent to recover in the post-anesthesia care unit (PACU).

PACU staff monitor the patient's overall condition before discharge or transfer. Though anesthesia providers are present, most staffing typically consists of Registered Nurses (RNs), and the provider to patient ratio is lower relative to the operating room (OR). As a result, the PACU is less equipped to deal with airway complications. Critical incidents can prolong patients' length of stay (LOS), which in turn disrupts the OR schedule, creating back-ups and inefficiencies in resource utilization [2–4]. Desaturation events are common and of particular concern; up to 26.3% of hypoxemic events in the PACU may remain unresolved for over 10 min, leading to increased risks of future clinical deterioration [5, 6].

Though literature regarding desaturation in the PACU exists, it is lacking in the context of deep extubation. Thus, this study describes the occurrence of desaturation (SpO2 < 90%, > 10 s) following deep extubation. Our objective was to determine whether different case factors contribute to the incidence of desaturation and the effect of desaturation on time spent in PACU recovery.

The data presented here is an extension of the research article by Juang et al. on the Incidence of Airway Complications Associated with Deep Extubation in Ambulatory Ophthalmic and Head-and-Neck Surgery in Adults [7].

## Main text

### Materials and methods

#### Study population

This study used data from the single-arm, unblinded, observational deep extubation study by Juang and colleagues. [7]. Briefly, a total of three hundred patients over eighteen years old were enrolled over six months with no specific exclusion criterion. Data were collected towards the end of surgery on patients identified by a research coordinator as deep extubation candidates. These patients were followed from the end of surgery (in the OR) to PACU discharge for data collection.

#### Ethics

The study was approved by the Institutional Review Board (IRB) of Massachusetts Eye and Ear Infirmary and was registered at Clinicaltrials.gov. As the research study involves no more than minimal risk to patients, the IRB waived the need to obtain written informed consent.

#### Data collection

Patient age, sex, BMI, type of surgery, ASA PS classification, Mallampati's class, respiratory tract pathology and infection, intubation record, and PACU times were extracted from an electronic medical record. Detailed PACU desaturation events (defined by SpO2 < 90% for longer than 10 s) and vital signs were collected prospectively by the research coordinator.

#### Statistical analysis

Patients were classified into two groups: those with desaturation events in the PACU and those without. Patient demographics, characteristics, procedure types, intubation records, and intraoperative variables were analyzed. Statistical analyses were performed using SAS software version 9.4 (SAS Institute, Cary, NC). Continuous variables were summarized using median and interquartile ranges (q1-q3), while categorical variables were summarized using frequencies and percentages. Wilcoxon rank-sum tests were used to compare continuous variables among groups. Chi-square tests (Fisher's exact test for small samples) were used to compare categorical variables among groups.

### Results

A total of 300 patients were examined in this study. Desaturation, as defined in the data collection section under materials and methods, was observed in 13 out of 300 (4.3%) patients.

When comparing patient demographics between the desaturation and the no-desaturation groups, no significant difference was found in age, sex, and BMI. Similarly, no significant difference was found when comparing the type of surgery (ear, eye, neck, nose, throat, thyroid), ASA PS classification, Mallampati's class, and Cormack and Lehane's grade.

History of asthma was found to be comparatively noteworthy, with the desaturation group having a significantly larger percentage of patients with asthma than the no-desaturation group (46.15% vs. 17.77% p = 0.0210). The no-desaturation group, as compared to the desaturation group, was disproportionately composed of patients with no history of respiratory tract pathology (61.32% vs. 23.08%, p = 0.0082). No other respiratory pathology tested (Chronic cough, OSA, recent respiratory tract infection, chronic obstructive pulmonary disease (COPD), current smoker) was found to be statistically significant.

Prevalence of comorbidities (none, diabetes, GI obstruction, hiatal hernia, gastroesophageal reflux disease (GERD)), Laryngoscopy device used (Glidescope, Macintosh blade, Miller blade, Other), and the number of direct Laryngoscopy attempts were not significantly different when comparing the desaturation and no-desaturation groups.

Finally, there was no significant difference in time spent in phase 1 PACU (75 vs. 70 min, p = 0.3253) nor phase 2 PACU (59.0 vs. 74.5 min, p = 0.3549) when comparing the desaturation and the no-desaturation groups.

### Discussion

Desaturation events are a major concern in the PACU, leading to time and resource inefficiencies, and potentially more severe postoperative respiratory complications [8, 9]. As such, we chose it as the primary metric to judge the viability of deep extubation after release from the OR. The prevalence of desaturation (SpO2 < 90%, > 10 s) in adults in the PACU was found to be 4.3%. Interestingly, no significant difference was observed in Phase 1 and Phase 2 PACU recovery time. A probable explanation for this phenomenon is that all instances of desaturation lasted for less than a minute, and drastic, time-consuming interventions such as reintubation were neither necessary nor performed.

The 4.3% rate observed in our study is lower, but within the range of a previous study by Kaushal et al., which reported a desaturation incidence of 13.5% [10]. It is also consistent with an investigation by Smith et al., who reported a range of 1.1–13.3% [11]. The difference in study methods may account for the higher rates reported. These studies utilized a lower alarm limit of ≤ 94%, while we defined desaturation as < 90%. In Smith's study, no patients were found to have a SpO2 < 90%. Additionally, in both of these studies, patients remained on room air until desaturation, while the providers in our study were able to preemptively intervene as they saw fit.

While most literature discusses COPD as the primary indication of potential desaturation, our study found asthma to be the only statistically significant condition. While asthma is traditionally considered reversible when compared to COPD, recent studies suggest that COPD exhibits moderate reversibility when treated with anticholinergics or β2 agonists [12]. It must also be noted that COPD and recent respiratory tract infection, while not statistically significant, had p-values of 0.0642 and 0.0849. The number of patients in the study with a history of respiratory tract pathology was small, so further investigation may be warranted.

Our findings indicate that deep extubation is associated with a low rate (4.3%) of desaturation in the care of an experienced PACU team. Combined with similar PACU recovery times for both patients who did and did not experience desaturation, our data suggest that deep extubation for patients without significant respiratory tract pathology is a safe and viable alternative to traditional awake endotracheal extubation.

## Limitations

1. The study could not be blinded as the anesthesiologists and research staff are required to manage and observe the deep extubation technique.
2. Since none of the patients with a history of difficult airway underwent deep extubation due to safety concerns could lead to inherent selection bias
3. Experienced PACU nursing providers remained with each patient until adequate control of the airway was achieved, contributing to the low incidence rate of desaturationc (Table 1).

**Table 1:** Comparison of patient and case characteristics in the Post-Anesthesia Care Unit (PACU) between desaturation and no-desaturation groups. Data are expressed as median (q1-q3)

Characteristics	Total (n = 300)	PACU Desaturations		P-value
		Yes (n = 13, 4.3%)	No (n = 287, 95.7%)	
Age, median (q1–q3)	50.0 (31.0–61.5)	38.0 (29.0–55.0)	50.0 (31.5–62.0)	0.1569
Male, n (%)	139 (46.3%)	6 (46.15)	133 (46.34)	0.9894
Body mass index in kg/m2, median (q1-q3)	26.4 (23.1–29.8)	28.0 (25.3–32.7)	25.9 (23.0–29.8)	0.1755
Surgery class				
Ear	53 (17.7)	3 (23.08)	50 (17.42)	0.0890
Eye	15 (5.0)	0 (0.0)	15 (5.23)	
Neck	50 (16.7)	0 (0.0)	50 (17.42)	
Nose	87 (29.0)	8 (61.54)	79 (27.53)	
Throat	73 (24.3)	1 (7.69)	72 (25.09)	
Thyroid	22 (7.3)	1 (7.69)	21 (7.32)	
ASA				
1	65 (21.7)	4 (30.77)	61 (21.25)	0.7288
2	208 (69.3)	8 (61.54)	200 (69.69)	
3	27 (9.0)	1 (7.69)	26 (9.06)	
Mallampati's class (n = 295)				
1	114 (38.6)	4 (30.77)	110 (39.01)	0.7576
2	142 (48.1)	8 (61.54)	134 (47.52)	
3	36 (12.2)	1 (7.69)	35 (12.41)	
4	3 (1.0)	0 (0.0)	3 (1.06)	
	1 (0.3)	0 (0.0)	1 (0.35)	1.0000
Respiratory tract pathology history				
None	179 (59.7)	3 (23.08)	176 (61.32)	0.0082*
Asthma	57 (19.0)	6 (46.15)	51 (17.77)	0.0210*
Chronic cough	1 (0.3)	0 (0.0)	1 (0.35)	1.0000
OSA	29 (9.7)	2 (15.38)	27 (9.41)	0.3636
Snoring	33 (11.0)	2 (15.38)	31 (10.80)	0.6422
Recent respiratory tract infection within past 4 weeks	2 (0.7)	1 (7.69)	1 (0.35)	0.0849
COPD	10 (3.3)	2 (15.38)	8 (2.79)	0.0642
Current smoker	26 (8.7)	2 (15.38)	24 (8.36)	0.3129
Cormack and Lehane's grade (n = 298)				
1	245 (82.2)	11 (84.62)	234 (82.11)	1.0000
2	48 (16.1)	2 (15.38)	46 (16.14)	
3	4 (1.3)	0 (0.0)	4 (1.40)	
4	1 (0.3)	0 (0.0)	1 (0.35)	
Difficulty intubation	0 (0.0)	0 (0.0)	0 (0.0)	
Comorbidity				
None	220 (73.3)	9 (69.23)	211 (73.52)	0.7516
Diabetes	25 (8.3)	1 (7.69)	24 (8.36)	1.0000
GI obstruction	0 (0.0)	0 (0.0)	0 (0.0)	
Hiatal Hernia	2 (0.7)	0 (0.0)	2 (0.70)	1.0000
GERD	63 (21.0)	4 (30.77)	59 (20.56)	0.4830
Laryngoscopy device used				
Glidescope	51 (17.0)	2 (15.38)	49 (17.07)	1.0000
MAC	236 (78.7)	11 (84.62)	225 (78.40)	0.7421
Miller	12 (4.0)	0 (0.0)	12 (4.18)	1.0000
Other	0 (0.0)	0 (0.0)	0 (0.0)	
Number of direct laryngoscopy attempts (n = 293)				
1	277 (94.5)	13 (100.00)	264 (94.29)	1.0000
2	13 (4.4)	0 (0.0)	13 (4.64)	
3	3 (1.0)	0 (0.0)	3 (1.07)	
Phase 1 PACU in min, median (q1–q3), n = 269	70 (58–88)	75 (59–118)	70 (58–87)	0.3253
Phase 2 PACU in min, median (q1–q3), n = 222	74 (58–101)	59.0 (48.3–101.0)	74.5 (58.3–100.8)	0.3549

* Statistical significance with P-value < 0.05

## Data Availability

The datasets used and analyzed during the current study available from the corresponding author on reasonable request.

## Abbreviations

ASA PS: American Society of Anesthesiologists Physical Status
BMI: Body Mass Index
COPD: Chronic obstructive pulmonary disease
FiO2: Fraction of inspired oxygen
GA: General anesthesia
GERD: Gastroesophageal reflux disease
IRB: Institutional review board
LOS: Length of stay
MEEI: Massachusetts eye and ear infirmary
OR: Operating room
PACU: Post-Anesthesia Care Unit
RN: Registered nurses
SpO_2_: Saturation of peripheral oxygen
